# Microinvasive cervical squamous cell carcinoma in Slovenia during the period 2001–2007

**DOI:** 10.2478/raon-2014-0010

**Published:** 2014-07-10

**Authors:** Helena Gutnik, Jasenka P. Matisic, Maja Primic Zakelj, Margareta Strojan Flezar

**Affiliations:** 1 Institute of Pathology, Faculty of Medicine, University of Ljubljana, Slovenia; 2 British Columbia Cancer Agency, Vancouver, Canada; 3 Epidemiology and Cancer Registry, Institute of Oncology Ljubljana, Slovenia; 4 Institute of Pathology, Faculty of Medicine, University of Ljubljana, Slovenia

**Keywords:** cervical cancer, cervical squamous cell carcinoma, microinvasive squamous cell carcinoma, cervical intraepithelial neoplasia

## Abstract

**Background:**

Microinvasive squamous cell carcinoma (MISCC) comprises a significant portion of all cervical cancers in Slovenia. Criteria of carcinomatous invasion are well described in the literature, however histopathological assessment of MISCC is difficult, because morphological characteristics can overlap with cervical intraepithelial neoplasia grade 3 (CIN 3) and other pathological changes. The aim of our study was to evaluate the reliability of the histopathological diagnosis of MISCC in Slovenia during the period from 2001 to 2007.

**Materials and methods.:**

Data on patients with a histopathological diagnosis of cervical MISCC (FIGO stage IA) in the period of 2001 to 2007 were obtained from the Cancer Registry of Slovenia. Histological slides were obtained from the majority of pathology laboratories in Slovenia. We received 250 cases (69% of all MISCC) for the review; 30 control cases with CIN 3 and invasive squamous cell carcinoma FIGO stage IB were intermixed. The slides were coded and reviewed.

**Results:**

Among 250 cases originally diagnosed as MISCC, there was an agreement with MISCC diagnosis in 184 (73.6%) cases (of these 179/184 (97.3%) cases were FIGO stage IA1 and 5/184 (2.7%) cases were FIGO stage IA2). Among 179 FIGO stage IA1 cases 117 (65.4%) showed only early stromal invasion.

**Conclusions:**

The retrospective review of cases diagnosed as MISCC during the period 2001–2007 in Slovenia showed a considerable number of overdiagnosed cases. Amongst cases with MISCC confirmed on review, there was a significant proportion with early stromal invasion (depth of invasion less than 1 mm).

## Introduction

**FIGURE 1.**
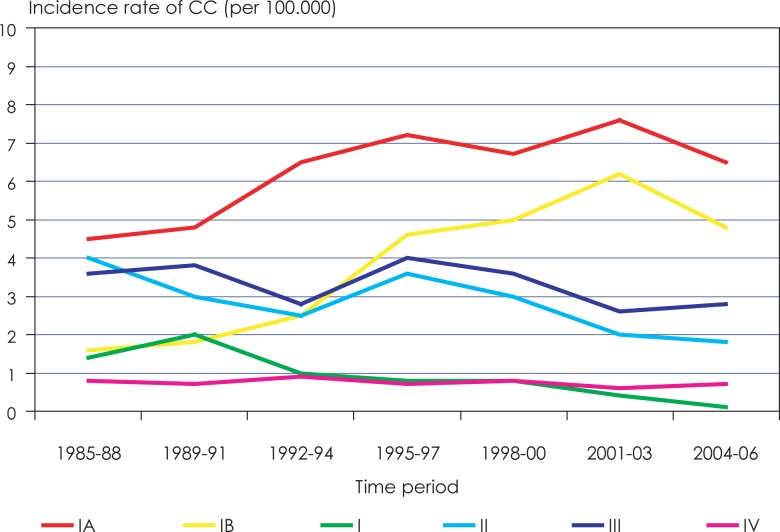
Incidence rate of cervical cancer according to the clinical stage at the diagnosis (Slovenia 1985–2006). *CC* = cervical carcinoma; *IA, IB, I, II, III, IV*: clinical stages of cervical cancer according to international federation of gynecology and obstetrics (FIGO) (Source: Report on results of the national cervical cancer screening programme ZORA (in the period 2007–2008))

**FIGURE 2.**
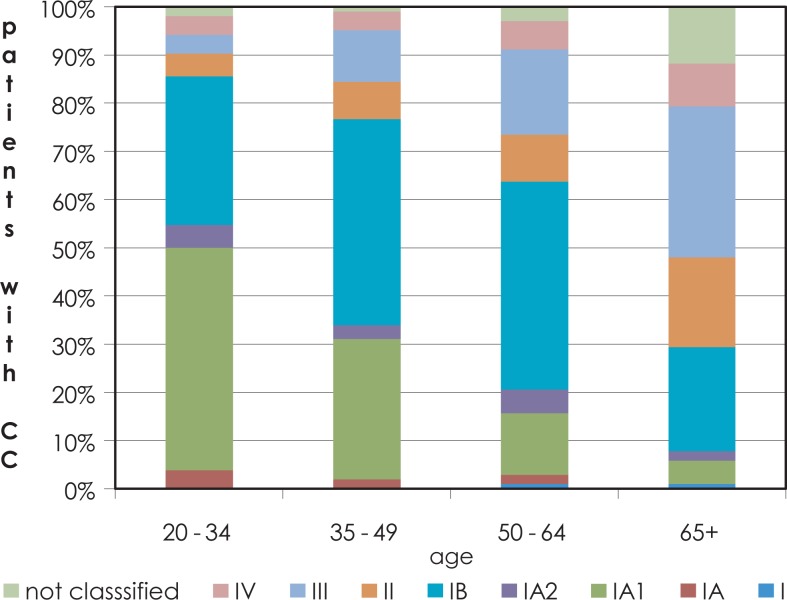
Proportion of patients with cervical carcinoma by clinical stage of International Federation of Gynaecology and Obstetrics (FIGO) and by age groups (Slovenia 2004–2006). *CC* = cervical carcinoma; *IA, IA1, IA2, IB, II, III, IV*: clinical stages of cervical carcinoma according to the International Federation of Gynecology and Obstetrics (FIGO) (Source: Report on results of the national cervical cancer screening program ZORA (in the period 2007–2008))

For more than 50 years, the Cancer Registry of the Republic of Slovenia has collected epidemiological data on the incidence rate of cervical cancer and cervical intraepithelial neoplasia (CIN). The incidence rate of cervical cancer was declining until the late 1980s, however it began to rise by the beginning of the 1990s and exceeded 20 new cases per 100.000 women (the highest incidence rate was 23.0/100.000 in 1997).^1^–^4^ During the same time period, incidence of the earliest stage of cervical squamous cell carcinoma, microinvasive (early invasive) squamous cell carcinoma (MISCC), increased and contributed substantially to overall cervical cancer incidence (Figure 1).^1^–^4^ The incidence of MISCC was highest in the age group 20–49 years. In the time period 2004–2006 it even exceeded 40% of all cervical cancer cases in this age group (Figure 2). Of the two FIGO clinical stages of MISCC the incidence of stage IA1 (tumor invasion 3 mm or less in depth) was much higher than the incidence of stage IA2 (tumor invasion 5 mm or less in depth) (Figures 1, 2).^5^ After the implementation of an organized national cervical cancer screening program in Slovenia in 2003 named *Z*godnje *O*dkrivanje pred*RA*kavih sprememb materničnega vratu (*ZORA*) the incidence of cervical cancer started to decline in 2004 with a concurrent rise in incidence of cervical intraepithelial neoplasia grade 3 (CIN 3).^3^ In the year 2007 the number of newly detected cervical cancers declined to 153 (incidence rate 14.8/100.000) and in 2008 and 2009 to 130 (incidence rate 12.6/100.000). Simultaneously the incidence rate of CIN 3 rose from 60.0/100.000 in 1997 to 115.0/100.000 in 2007.^3^

In many cervical biopsies light-microscopic evaluation of MISCC is a difficult task. It is partially the consequence of the anatomical and histological peculiarities of the cervix uteri, namely the tendency of CIN 3, from which the MISCC foci derive, to involve the cervical gland crypts.^6^,^7^ On the other hand histopathological changes linked to inflammation, prior biopsies, cautery artefacts and tangential sectioning of epithelium may mimic the MISCC.^6^,^7^ The experienced pathologist should consider all the options and carefully confirm or exclude them to avoid overdiagnosing invasive disease.^6^–^10^

The earliest form of MISCC is early stromal invasion, with the depth of invasion up to 1 mm. Early stromal invasion is encompassed in the IA1 FIGO clinical stage of cervical squamous carcinoma and was never staged as a separate category.^5^,^11^,^12^ Some authors think early stromal invasion should be considered as a separate category excluded from IA1 FIGO clinical stage because its management and prognosis is similar to that of CIN 3.^13^,^14^

Some authors describe the impact of biopsy management (different sectioning) and the number of histological levels per tissue block made on the evaluation of MISCC. According to the results of two studies, MISCC was present in the first tissue level in the majority of biopsies and that suggested that the processing of many hematoxylin eosin (HE) slides per tissue block causes an unnecessary burden for the pathologist.^15^,^16^

The purpose of our study was to find out whether the recorded incidence of cervical cancer in Slovenia in the period 2001–2007 was correct or overestimated resulting from difficulties implementing the histopathological criteria of MISCC and the existence of many histopathological changes that can mimic MISCC.

We also evaluated the presence of MISCC in the first tissue level and whether or not the number of HE slides per tissue block influenced the reliability of histopathological evaluation.

## Materials and methods

The study was approved by Slovenian national medical ethics committee (Approval No. 51/05/09). The data on microinvasive cervical squamous cell carcinoma (FIGO stage IA1 and IA2) in the period 2001–2007 were obtained from the Cancer Registry of RS. Original histological HE stained slides of cone biopsies and hysterectomies from which the original diagnoses of MISCC were made were requested from various pathology laboratories. In the period 2001–2007 eleven laboratories reported MISCC to the Cancer Registry of RS and 363 patients were diagnosed with MISCC. We managed to obtain 285 specimens from nine laboratories representing 78.5% of patients with the diagnosis of MISCC. After checking the HE slides 35 biopsies were missing or damaged and therefore excluded. The remaining 250 biopsies (69% of patients with the diagnosis of MISCC) were suitable for light microscopic review. The original diagnoses of 250 biopsies were: 230 carcinomas FIGO stage IA1, 16 carcinomas FIGO stage IA2, and 4 MISCC without stage evaluation.

To avoid bias in review, 30 control cone biopsies were included in the study (15 with the original histopathological diagnosis of CIN 3 and 15 with the original histopathological diagnosis of cervical squamous cell carcinoma, FIGO stage IB (SCC, IB)). The control biopsies were selected from the archives of the Institute of Pathology, Faculty of Medicine Ljubljana and from Department of Pathomorphology, Division of Obstetrics and Gynecology, University Medical Centre Ljubljana. Overall the study included 280 biopsies (274 cervical cone biopsies and 6 hysterectomies). The cases were anonymized and assigned randomly chosen successive numbers from 1 to 280.

**FIGURE 3.**
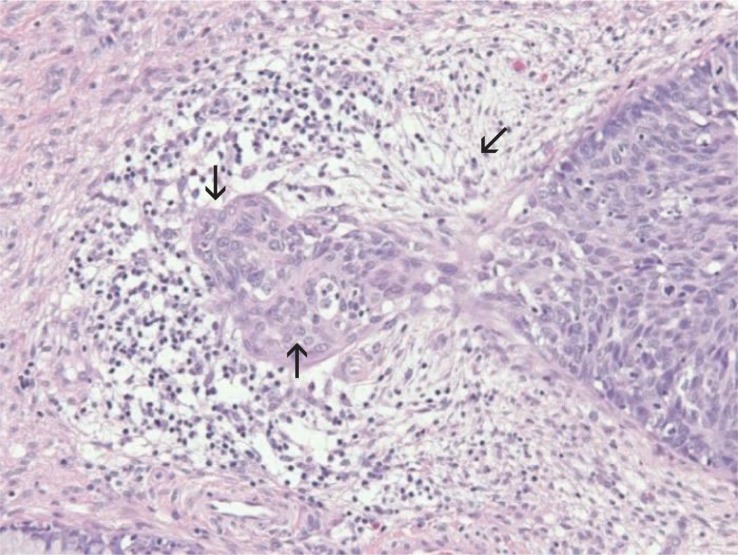
Focus of cervical microinvasive squamous cell carcinoma (left) emerging from cervical intraepithelial lesion grade 3 (right); maturation of the neoplastic epithelium (↑), irregularities of the epithelial stromal interface (↓) and desmoplastic reaction in the adjacent stroma (↙) are seen (hematoxylin and eosin stain; original magnification: ×200).

Firstly the HE slides were reviewed independently by two pathologists (pathologist A, pathologist B) experienced in the histopathological diagnosis of uterine cervical lesions. Then third pathologist (pathologist C) independently, without knowing the original diagnoses or diagnoses of pathologists A and B, reviewed selected cases including those where the first two pathologists did not find any MISCC, the biopsies where they disagreed, the biopsies where the histopathological changes were difficult to interpret but were considered suspicious for MISCC, and some randomly chosen control biopsies. The established criteria for histopathological evaluation of micro-invasive cervical squamous cell carcinoma were used: focal maturation of the neoplastic epithelium with prominent nucleoli, desmoplastic response in the adjacent stroma, blurring and irregularity of the epithelial-stromal interface (Figure 3).^6^,^7^,^10^,^11^ The depth and width of MISCC was measured in millimeters (mm) and classified according to the FIGO classification of cervical squamous carcinoma clinical stages.^5^ Every foci of invasion were measured separately from the CIN 3 epithelial-stromal interface. When the pathologists could not confirm the diagnosis of microinvasive carcinoma, they also assessed which histopathological changes that were present in the biopsy could mimic early invasion.

The results of the review were compared to the original diagnoses and presented with the methods of descriptive statistics. The interobserver variability between the three pathologists was measured by Cohen’s kappa coefficient. The diagnosis of MISCC was confirmed when two or all three pathologists agreed on diagnosis.

Because the data on the type of cone biopsy and tissue management in the histopathology laboratories were not available for all patients, we also recorded the type of cone biopsy (cold knife or large loop excision of the transformation zone (LLETZ)) and the method of tissue sectioning (parallel or radial). The average number of tissue levels per tissue block was also counted.

In all biopsies with the review assessment of MISCC we evaluated the presence of MISCC in the first level cut from the tissue block.

## Results

The average age of patients with an original diagnosis of microinvasive carcinoma was 41.1 years (range 22–74 years, median 40). The average age of patients from the control group with the CIN 3 diagnosis was 35.9 years (range 24–55 years, median 40) and from the control group with the diagnosis of SCC, IB was 39.5 years (range 31–51 years, median 40). Among all 280 biopsies included in the study, 196 (70%) were cold-knife cones, 78 (28%) were LLETZ, and 6 (2%) were hysterectomy specimens. There were 269 (96%) cones sectioned in the parallel way, and 5 cones and 6 hysterectomies (4%) sectioned using the radial method. The average number of tissue levels per tissue block was 8 (range 1–40).

The results of the review by pathologists A and B were: among 250 biopsies with an original MISCC diagnosis 184 biopsies were confirmed as MISCC (31 as suspicious for MISCC). In 50 biopsies the reviewers agreed there was no MISCC. In 15 biopsies the agreement could not be reached. 1 biopsy was assessed as SCC, stage IB by both pathologists. All CIN 3 control biopsies were confirmed as CIN 3. Among SCC, FIGO stage IB control biopsies the diagnosis of SCC, FIGO stage IB was confirmed in 12 cases. However, 3 biopsies were evaluated as MISCC, 2 as FIGO stage IA1 and 1 as FIGO stage IA2. Cohen’s kappa coefficient for pathologists A and B diagnostic agreement was 0.85.

Pathologist C evaluated 92 biopsies (32.8%): all 15 biopsies where pathologists A and B could not reach an agreement, 42 biopsies where pathologists A and B agreed there was no MISCC, 31 biopsies where pathologists A and B thought they were difficult to interpret and agreed on suspicious of MISCC diagnosis and 4 randomly chosen control biopsies. The Cohen’s kappa coefficients for this group of biopsies (with the original diagnosis of MISCC), reviewed by all three pathologists were: 0,80 for pathologists A and B, 0,80 for pathologists A and C and 0,88 for pathologists B and C (Table 1). We considered the case as MISCC when two or all three pathologists agreed on this diagnosis. The 31 cases that pathologists A and B found difficult to interpret were reexamined concurrently by all three pathologists to reach a consensus diagnosis.

The final results of the three pathologists, review are shown in Table 2. Among 184 biopsies with confirmed MISCC 179 were defined as FIGO stage IA1 (117 early stromal invasion) with maximum depth of invasion of 1 mm, 62 with depth of invasion between 1 and 3 mm) and 5 biopsies were defined as FIGO stage IA2.

**FIGURE 4.**
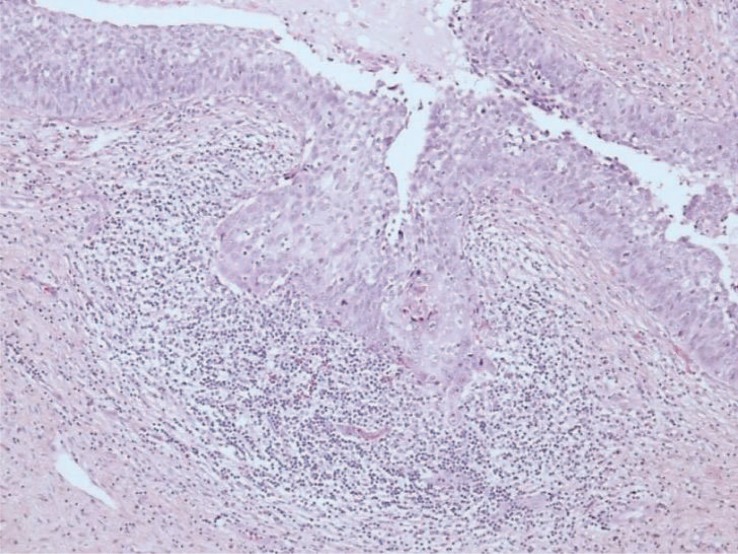
Heavy inflammatory infiltrate surrounding the focus of invasion; cell maturation and scalloped margins are present but no convincing desmoplasia. (hematoxylin and eosin stain; original magnification: ×200).

The major histopathological criteria of early invasion were evaluated in all 187 biopsies scored as MISCC (184 with original MISCC diagnosis, 3 with original SCC, FIGO stage IB diagnosis). Focal maturation of the neoplastic epithelium with prominent nucleoli was confirmed in all 187 biopsies. Blurring and irregularities of the epithelial-stromal interface was seen in 159 (85%) of 187 biopsies. A desmoplastic response in the adjacent stroma was confirmed in 133 (71%) of 187 biopsies (in the remaining 54 biopsies a heavy mononuclear inflammatory infiltrate was surrounding invasive foci making the evaluation of desmoplastic reaction impossible) (Figure 4).

**FIGURE 5.**
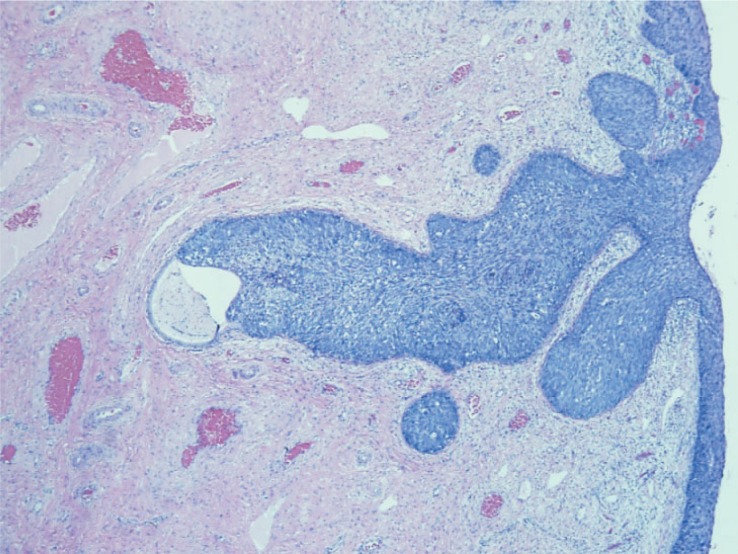
Cervical intraepithelial neoplasia grade 3 involving the cervical gland crypts (hematoxylin and eosin stain; original magnification: ×40).

In 64 biopsies evaluated as not containing MISCC we evaluated the histopathological changes that could possibly mimic early invasion and cause false overdiagnoses of MISCC. We classified these histopathological changes into eight groups: deposits of normal squamous or CIN epithelium in the subepithelial tissue, tangentially sectioned epithelium, prior biopsy site changes, reactive epithelial changes (regeneration, pseudoepithelomatous hyperplasia), inflammatory changes with blurred epithelial-stromal interface, complex CIN 3 with extensive gland-crypt involvement, cautery tissue artifacts, and normal squamous epithelium without evident pathological changes. The most frequent solitary histopathological change was complex CIN 3 with extensive gland crypt involvement (41 of 64 biopsies (64%)) followed by inflammatory changes and tangentially sectioned epithelium (Figure 5).

In 187 biopsies confirmed as MISCC after review (184 with an original diagnosis of MISCC, and 3 SCC, FIGO stage IB from the control group) we also evaluated the presence of MISCC in the first tissue level of the block. We found MISCC in first tissue level in 141 of 187 biopsies (75%). In the remaining 46 biopsies (25%) MISCC was present in the second or subsequent tissue levels. In 42 of 46 biopsies the absence of MISCC in the first tissue level was the consequence of a poorly prepared first cut HE slide with large areas of epithelium missing. MISCC was found in 2^nd^ level in 5/42 cases, 3^rd^ level in 12/42 cases, 4^th^ level in 8/42 cases, 5^th^ level in 5/42 cases, 6^th^ level in 4/42 cases, 7^th^ level in 6/42 cases, 8^th^ and 9^th^ level in one case each/42 cases. In only 4 of 46 biopsies no MISCC was found in suitably prepared first cut HE slide; MISCC was found in 2^nd^ level in 2/4 cases and 3^rd^ level in 2/4 cases.

## Discussion

Cervical cancer is still a significant public health problem even in some countries with successfully implemented cytological screening programs for early detection of cervical precancerous lesions. Due to the high incidence of cervical carcinoma in Slovenia and the especially high incidence of MISCC, we tried to assess, the reliability of the histopathological diagnosis of MISCC during the period 2001–2007.^1^–^4^

Our study exposes several problems in the diagnosis of MISCC. 25.6% of biopsies with an original diagnosis of MISCC were assessed as not containing MISCC after review. Among the biopsies assessed as MISCC after the review, 63.6% were evaluated as early stromal invasion. In the majority of biopsies the established criteria of early invasion were readily assessable with the exception of the desmoplastic reaction which could not be reliably assesed due to heavy inflammatory infiltrate in 29% of cases. In cases confirmed as MISCC, the MISCC was found in the first tissue level in only 75% of biopsies.

The rate of overdiagnosis of MISCC in our study (25.6%) is comparable to that found in large biopsy series reviews.^17^–^19^ The rate of overdiagnosis is much higher than of underdiagnosis of MISCC. These results show that when in doubt pathologists are prone to overdiagnose MISCC. Overdiagnosis of MISCC not only increases cervical cancer incidence rates but also exposes women to unnecessary overtreatment and is a cause of psychological and social stresses.^17^,^18^,^20^

We were unable to obtain epidemiologic data about the proportion of MISCC in the overall FIGO stage IA1 groups of cervical carcinoma in other countries, but according to the data of some published studies the incidence of MISCC is below the Slovenian incidence. Two separate studies reported that MISCC comprised 15% and 19% of cervical cancers.^19^,^21^ In our study we found out that among MISCC not only the percentage of FIGO stage IA1 is especially high (92% of original MISCC diagnoses and even 97% cases with MISCC confirmed after review) but also the early stromal invasion with invasive foci of less than 1 mm depth represented a high proportion of FIGO stage IA1 cases. To our knowledge cancer registries do not classify early stromal invasion as a separate category, although some authors think it should be excluded from MISCC because its prognosis and management are similar to those of CIN 3. In their opinion reporting early stromal invasion as invasive squamous carcinoma increases the incidence of cervical cancer and the cancer burden.^13^,^14^ We agree with this opinion which is supported by Slovenian epidemiological data that show that mortality from cervical cancer is low compared with its incidence rate.^2^–^4^,^22^

Regarding the evaluation of criteria for early invasion we had no substantial difficulties in assessing focal maturation with prominent nucleoli of the neoplastic epithelium and blurring and irregularities of the epithelial-stromal interface.^6^,^7^,^10^ However a desmoplastic response in the adjacent stroma was difficult or impossible to evaluate in 29% of all biopsies reevaluated as MISCC because of the heavy mononuclear inflammatory infiltrate surrounding and sometimes invading the foci of MISCC. We found no evidence of this problem in the literature, but the difficulties in assessing this criterion are disturbing because we believe that a clearly expressed desmoplastic reaction is a valuable criterion of early invasion.

We also estimated the presence of MISCC in first tissue level per block and compared the results of data from previous studies. Pathologists from different countries examine variable number of tissue levels per blocks of cone biopsies, depending on their national societies’ recommendations and laboratory protocols. In our study we noticed that the number of tissue levels varied from 1 to 40 levels from nine different pathology laboratories we received the biopsies from. This is the consequence of the fact that in Slovenia we are only now starting to prepare standard operating procedures to manage uterine cervical biopsies whereas previously pathology laboratories would follow their own recommendations. Nevertheless, the results of previous studies showed that MISCC was present in the first tissue level in the majority of the cone biopsies; therefore larger numbers of levels have been regarded as unnecessary except when there was discordance between cytologic, colposcopic and histologic data.^15^,^16^ The results of our present study do not confirm these previous findings, as we found MISCC in the first tissue level in only 75% of cone biopsies with a confirmed diagnosis of MISCC. The reason for this discordance is suboptimal quality of the first tissue level (first HE slide) preparation in some laboratories. In our opinion the first levels are often not suitable for pathological evaluation because of large areas of squamous epithelium missing. In previous studies this issue was not evident as the tissue levels were presumably suitable for evaluation. This fact emphasizes the need for proper recommendations and quality assurance for pathology laboratories. According to our study 5 tissue levels would assure diagnosis in all cases if the optimal quality of the first tissue level preparation was secured.

In conclusion, our findings suggest that diagnosis of MISCC is demanding and pathologists in their routine work tend to overdiagnose the invasive disease. This leads to increased cervical cancer incidence rates and also exposes women to unnecessary overtreatment. The results of our study show the need for national recommendations and quality assurance at all levels of uterine cervical diagnostics related to national cervical screening program.

## Figures and Tables

**TABLE 1.** Results of review of 88 biopsies with original diagnosis of cervical microinvasive squamous cell carcinoma examined by three pathologists (Pathologist A, Pathologist B, Pathologist C)

CKC		B +	B –		A +	A –		B +	B –
	A +	31	10	C +	28	3	C +	28	3
	A –	4	43	C –	13	44	C –	7	50
	0,80			0,80			0,88		

CKC = Cohen s kappa coefficient; *+* = diagnosis of microinvasive squamous cell carcinoma confirmed; – = diagnosis of microinvasive squamous cell carcinoma not confirmed; A = Pathologist A; B = Pathologist B; C = Pathologist C

**TABLE 2.** Final results of review on 280 biopsies with original diagnosis of cervical microinvasiove squamous cell carcinoma: 250 biopsies with the original diagnosis of cervical microinvasive squamous cell carcinoma and 30 control biopsies

	**Number of biopsies**	**Original diagnosis**	**Diagnosis after the review N (%)**
Control biopsies	15	CIN 3	15 (100,0) CIN 3
Control biopsies	15	SCC IB	12 (80,0) SCC IB 3 (20,0) SCC IA
Biopsies to reevaluate (with the original MISCC diagnosis)	250	MISCC	184 (73,6) SCC IA 2 (0,8) SCC IB 64 (25,6) no MISCC
All	280		280

MISCC = microinvasive squamous cell carcinoma; IA, IB = clinical stages of cervical squamous cell carcinoma according to International Federation of Gynecology and Obstetrics (FIGO) classification; CIN 3 = cervical intraepithelial lesion grade 3; SCC = squamous cell carcinoma
